# Imaging Proteolytic Activity in Live Cells and Animal Models

**DOI:** 10.1371/journal.pone.0066248

**Published:** 2013-06-11

**Authors:** Stefanie Galbán, Yong Hyun Jeon, Brittany M. Bowman, James Stevenson, Katrina A. Sebolt, Lisa M. Sharkey, Michael Lafferty, Benjamin A. Hoff, Braeden L. Butler, Susan S. Wigdal, Brock F. Binkowski, Paul Otto, Kris Zimmerman, Gediminas Vidugiris, Lance P. Encell, Frank Fan, Keith V. Wood, Craig J. Galbán, Brian D. Ross, Alnawaz Rehemtulla

**Affiliations:** 1 Center for Molecular Imaging, the University of Michigan Medical School, Ann Arbor, Michigan, United States of America; 2 Department of Radiation Oncology, the University of Michigan Medical School, Ann Arbor, Michigan, United States of America; 3 Cellular and Molecular Biology Program, the University of Michigan Medical School, Ann Arbor, Michigan, United States of America; 4 Department of Biological Chemistry, the University of Michigan Medical School, Ann Arbor, Michigan, United States of America; 5 Department of Radiology, the University of Michigan Medical School, Ann Arbor, Michigan, United States of America; 6 Promega, Madison, Wisconsin, United States of America; Stanford University, United States of America

## Abstract

In addition to their degradative role in protein turnover, proteases play a key role as positive or negative regulators of signal transduction pathways and therefore their dysregulation contributes to many disease states. Regulatory roles of proteases include their hormone-like role in triggering G protein-coupled signaling (Protease-Activated-Receptors); their role in shedding of ligands such as EGF, Notch and Fas; and their role in signaling events that lead to apoptotic cell death. Dysregulated activation of apoptosis by the caspase family of proteases has been linked to diseases such as cancer, autoimmunity and inflammation. In an effort to better understand the role of proteases in health and disease, a luciferase biosensor is described which can quantitatively report proteolytic activity in live cells and mouse models. The biosensor, hereafter referred to as GloSensor Caspase 3/7 has a robust signal to noise (50–100 fold) and dynamic range such that it can be used to screen for pharmacologically active compounds in high throughput campaigns as well as to study cell signaling in rare cell populations such as isolated cancer stem cells. The biosensor can also be used in the context of genetically engineered mouse models of human disease wherein conditional expression using the Cre/loxP technology can be implemented to investigate the role of a specific protease in living subjects. While the regulation of apoptosis by caspase's was used as an example in these studies, biosensors to study additional proteases involved in the regulation of normal and pathological cellular processes can be designed using the concepts presented herein.

## Introduction

Development of new agents that can be used to treat a wide variety of human diseases including cancer, stroke, cardiovascular and neurodegenerative diseases such as Alzheimer's and Parkinson's continues unabated. While for example, anticancer agents are designed as inducers of cell death for tumor cell eradication, anti-neurodegenerative agents are sought to ameliorate neuronal dropout through inhibition of cell death processes. New therapies universally undergo evaluation using cell-based assays followed by efficacy studies using appropriate mouse models. The variety and complexity of signaling events associated with cell death programs which can lead to mitotic catastrophe, apoptosis, necrosis, necroptosis, pyrosis and autophagy exemplify the importance of developing generalizable biomarker readouts of the cell death process. In an effort to assess and quantify efficacy of drug interventions, assays distinguishing induction and inhibition of signaling events have been developed predominantly for cell culture screens [1]. Caspase 3/7 activation is considered a key surrogate marker for assessment of apoptosis and the ability to image this process in intact cells and live animals has gained increasing interest particularly with the onslaught of molecularly targeted agents. Caspases mediate the early stages of apoptosis by proteolytically processing their substrates such as PARP thus their proteolytic function can be used in the design of radioactive, fluorescent and luminescent assays for assessing apoptosis [2].

While a variety of cell death assays have been used in high throughput screening campaigns [1], [3], [4] aiding in the rapid identification of efficacious therapies and uncovering drugable, dominant signaling pathways in cells, an assay with high sensitivity which can also be used for *in vivo* testing would be of significant benefit. In comparison to fluorescence based assays, those based on bioluminescence have significantly more sensitivity and wider dynamic range when used in library screens for small molecule modulators [5].

Our previously published Caspase 3 reporter, developed for *in vivo* bioluminescence imaging in living mice, utilized split luciferase technology in combination with strong interacting peptides to ensure enzyme reconstitution [6]–[9]. Briefly, separation of the monomeric luciferase enzyme into two components was achieved by an intervening Caspase 3 cleavage signal, DEVD. Caspase 3 activation led to cleavage of the DEVD sequence thereby releasing the two separate luciferase components to reconstitute activity assisted by the strong protein-protein interactions between Peptide A (PepA) and Peptide B (PepB), which were fused to both luciferase termini. While this approach was adequate for *in vivo* applications, HTS applications of this reporter were hindered by suboptimal background signal attributed to strong protein-protein interaction and intra-molecular binding of the reporter thus rendering it unsuitable for *in vitro* use.

In pursuit of a single and more sensitive Caspase reporter which could be used in both *in vitro* and *in vivo* applications, we significantly modified our initial reporter design to provide for an adaptable and highly sensitive imaging surrogate for Caspase activation which would be capable for applications in both HTS and *in vivo* bioluminescence imaging [6]–[9]. Using recent advances in protease biosensors including circularly permuted forms of firefly luciferase and thermal stable photinus pyralis luciferases [10] [5], we screened a library of random mutated thermal stable luciferase constructs permuted at residue 358 [11] and selected the best performing constructs for in depth testing. The resultant reporter was found to have excellent signal-to-noise characteristics required in HTS applications with robust fold induction (>50 fold) of bioluminescence activity following stimuli engaging receptor-mediated or intrinsic apoptotic pathway activation. Moreover, the exquisite sensitivity of the reporter allowed for evaluation of treatment strategies against rare subpopulations of isolated cancer stem cells. Furthermore, studies also revealed the reporter was effective for *in vivo* imaging of Caspase-induced cell death in mice bearing implanted xenograft tumors. Finally, the versatility of this reporter was extended into the development of a novel transgenic mouse, wherein Cre-recombinase mediated expression of the reporter confers broad applicability of the reporter across many models of human disease.

## Materials and Methods

### Ethics Statement

All animal experiments were performed in accordance with protocols approved by University Committee on Use and Care of Animals of the University of Michigan (UCUCA protocol # 08669 & 10412). Animals were housed in specific pathogen–free facilities at the University of Michigan. All surgery and imaging was performed under anesthesia using isofluorane, and all efforts were made to minimize suffering. Humane euthanasia of mice was performed by Carbon Dioxide with a flow rate of 20% of chamber volume/minute following UCUCA-approved animal research protocols.

### GloSensor (Caspase 3/7) vector construction and molecular evolution

A thermal stable variant of the luciferase from *Photuris pensylvanica* [11] was circularly permuted at residue 233 or 358 based on previous work using *Photinus pyralis* luciferase [5], [10]. For constructs permuted at residue 233, DNA encoding a Met-(233–544)-SS-DEVDG-SSG-(2–232)-Val fusion protein was cloned into the pF9A vector (Promega, WI). For constructs permuted at residue 358, DNA encoding a Met-(358–544)-S-DEVDG-SL-(4–354)-Val fusion protein was cloned into a derivative of pF9A modified for bacterial expression. The latter construct was used as a template for random mutagenesis by error prone PCR (GeneMorph II, Agilent, CA), and the resulting library was screened in bacterial lysates for increased sensor response following treatment with recombinant caspase 3. In total, 21,648 colonies containing on average 3 nt changes per clone were screened in *E. coli*, where 175 hits showing ≥20% increased response were confirmed in HEK293 cells using conditions similar to those of the GloSensor cAMP Assay (Promega, WI). In brief, 15,000 cells/well were plated in 96-well plates and transiently transfected using FuGENE HD (Promega, WI). After 24 hrs, medium was exchanged for CO_2_ independent medium (Life Technologies, NY) containing a 2% v/v dilution of the GloSensor cAMP Reagent (Promega, WI), and cells were allowed to equilibrate for two hours at room temperature. Following thermal equilibration in the luminometer, TRAIL ligand (1 µg/ml) was added and luminescence was monitored continuously for 10 hrs at 37°C. The best performing construct was identified as 358V3 which contained a single Thr to Ile point mutation at amino acid 151 (507 in the non-permuted construct) and was termed Caspase 3/7 GloSensor.

### Bioluminescence assay in cell culture

D54-MG (D54) and MDA-MB231/1833 (1833) cells were transfected with the Caspase 3/7 reporter using FuGENE reagent (Roche Diagnostics, Indianapolis IN, USA) according to manufacturer's protocol and placed in selection media (400 µg/ml G418, Invitrogen, Carlsbad CA, USA) 48 hrs post transfection. Single clones were selected and tested for reporter expression by western blotting with luciferase antibody (Promega, WI) and for bioluminescence. Clones with similar reporter expression and bioluminescence activity were selected for study. D54-MG glioma cells and MDA-MB231/1833 cells were maintained in RPMI media or DMEM, respectively, supplemented with 10% heat-inactivated FBS and 1% penicillin/streptomycin/glutamine (Gibco, Carlsbad CA, USA). Cells were grown in a humidified incubator at 37°C and 5% CO_2_.

Cells stably expressing the Caspase 3/7 reporter were seeded at a density of 1–2×10^4^ cells/well in a 96-well white clear bottom assay plate (Corning, Inc., Corning, NY, USA) 24 hrs prior to treatment. Cells were treated with 100–200 ng/ml TRAIL, 12.5 µM CV3988, 50 µM Docetaxel or 25–100 ng/ml anti-Fas antibody (EMD Millipore, Billerica MA) and imaged at indicated time points. Live-cell bioluminescent imaging was performed by adding 100 µg/ml of GloSensor cAMP reagent (Promega, WI) to the assay media. If cells were imaged at multiple time points media cells were incubated with 100 µl of CO_2_ independent media containing 300 µg/ml of GloSensor cAMP reagent (Promega, WI) for 2 hrs prior to treatment start. Photon counts were acquired over time pre- and post-treatment using the Envison luminometer (Perkin Elmer, Waltham MA, USA). For studies using the pan-Caspase inhibitor Z-VAD-FMK (R&D, Minneapolis MN) cells were pre-incubated with 20 µM Z-VAD-FMK or DMSO 1 hr prior to treatment start.

### High throughput screening (HTS)

Cells were seeded at a density of 1×10^4^ cells/well in a 96-well white clear bottom assay plate (Corning, Inc., Corning NY, USA) using automated pipettes. At 24 hrs post seeding, media was changed to CO_2_ independent media (Promega, WI) containing 1% GloSensor cAMP reagent (Promega, WI) (corresponds to 300 µg/ml GloSensor cAMP reagent in HEPES buffer) until a steady-state basal signal was obtained (2 hrs prior to start of treatment). Addition of media and compound library was performed using a Titertek Mulidrop Microplate Dispensor (Beckman NXP Laboratory Automation Workstation, Beckman Coulter, Fullerton, CA, USA). The LOPAC1280 compound library (Sigma-Aldrich, St. Louis, MO, USA) was used at final compound concentration of 10 µM in PBS. Fold changes in bioluminescence were calculated by normalizing values of compound treated wells to untreated wells every hr for a time of 24 hrs. Heat maps were generated using bioinformatics toolbox of Matlab software. The Z-factor was calculated as previously described: Zhang et al [12].
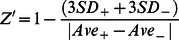
Where SD_+_ = positive control standard deviation, SD_−_ = negative control standard deviation, Ave_+_ = positive control average and Ave_−_ = negative control average. LOPAC1280 and CV3988 compounds were purchased from Sigma-Aldrich (St. Louis MO, USA). MNS and Docetaxel were purchased from TOCRIS bioscience (Ellisville MO, USA). Human recombinant TRAIL was made using his-tagged TRAIL vector as previously described [13].

### Western blot analysis and antibodies

Cells were seeded in 10 cm dishes and incubated with indicated drugs 24 hrs post seeding. Cells were washed with phosphate-buffered saline (PBS) and lysed with NP-40 lysis buffer (1% NP40, 150 mM NaCl, and 25 mM Tris, pH 8.0) supplemented with protease inhibitors (Complete Protease Inhibitor Cocktail, Roche) and phosphatase inhibitors (PhosSTOP, Roche). Concentration of protein was determined using Lowry assays (Bio-Rad, Hercules, CA) and equal amount of whole cell protein lysate was loaded in each lane and resolved using 4–12% gradient Bis-Tris gel (Invitrogen, CA). Proteins were transferred to 0.2 µm nitrocellulose membrane (Invitrogen, CA) or PVDF (Millipore). Membranes were incubated overnight at 4°C with primary antibodies after blocking, followed by incubation with appropriate HRP-conjugated secondary antibody at room temperature for one hour. ECL-Plus was used to detect the activity of peroxidase according to the manufacturer's protocol (Amersham Pharmacia, Uppsala, Sweden). Antibodies raised against full length or cleaved Caspase 3, cleaved PARP and CD133 antibodies were purchased from Cell Signaling Technology (Beverly MA, USA), anti-luciferase antibody, Promega (Madison, WI, USA) Anti-beta actin (conjugated with HRP) antibody was purchased from Abcam (Cambridge, MA, USA). Secondary HRP- and Cy5-labeled IgG antibodies were purchased from Jackson ImmunoResearch (St. Louis MO, USA).

### Establishment of xenograft models and in vivo bioluminescence imaging

All animal experiments were performed in accordance with protocols approved by the University Committee on Use and Care of Animals of the University of Michigan (UCUCA protocol # 08669 & 10412). Animals were housed in specific pathogen–free facilities at the University of Michigan. To establish flank xenograft models 2×10^6^ D54-MG cells stably expressing the Caspase 3/7 bioluminescent reporter were implanted subcutaneously into NOD/SCID mice (Charles River, Wilmington MA) and treatment was initiated when tumors reached approximately 100 mm^3^, as assessed by caliper measurements. *In vivo* studies were also accomplished using a breast cancer bone metastasis model wherein MDA-MB231/1833 cells stably expressing the Caspase 3/7 GloSensor were implanted into the tibia of SCID mice. Tumor growth was followed by MRI and treatment initiated when tumor reached 5–15 mm^3^.

For *in vivo* bioluminescence imaging, mice were anesthetized using a 1–2% isofluorane/air mixture and injected with a single i.p. dose of 150 mg/kg VivoGlo Luciferin (Promega, WI). Consecutive images were acquired before and 6 hrs post-treatment or as indicated in figures using an IVIS® imaging system (Perkin Elmer, Waltham MA). Fold induction of bioluminescence activation was calculated by normalizing post-treatment values to pre-treatment values of each individual animal.

### Generation of Caspase bioluminescence transgenic reporter mice

CMV-Cre (The Jackson Laboratory, Bar Harbor, ME) or p48Cre (Ptf1a-Cre) mice [14] (provided by Chris Wright Vanderbilt University, Nashville, Tennessee, USA) were intercrossed with the transgenic reporter mice, which were generated by the transgenic core facility of the University of Michigan using pro-nuclear microinjection of the transgene containing the apoptosis reporter into fertilized eggs obtained from FVB/N females. The transgenic construct was generated by cloning the Caspase 3/7 GloSensor into the pCLEX vector [15]. A constitutively active CAG promoter was used to drive expression of a green fluorescent protein (EGFP) to determine expression of the transgene is various tissues. The EGFP coding sequence was flanked by loxP sites and a polyA signal with a strong termination sequence preventing the transcription of the downstream located bioluminescent reporter construct. Upon Cre-mediated recombination, driven by a CMV promoter or the p48Cre promoter, the EGFP cassette would be excised and transcription of the bioluminescent reporter is irreversibly activated in Cre-expressing cells.

Mice were genotyped using the following primer: CMV-Cre forward oIMR1084 5′-GCGGTCTGGCAGTAAAAACTATC-3′ and reverse oIMR1085 5′-GTGAAACAGCATTGC TGTCACTT-3′; p48Cre forward p48 GT-Cre 5′-CATGCTTCATCGTCGGTCC-3′ and reverse p48 GT-Cre 5′-GATCATCAGCTACACCAGAG-3′; Apoptosis reporter mouse forward 358 F 5′-AGTTTCAACAGCCAAATGG-3′ and reverse 358 R 5′-CCGGAATAGCTGCATAAC GAGAT-3′.

### Induction of pancreatitis

Mice were subjected to two series of 6 hourly intraperitoneal injections of cerulein (Sigma-Aldrich, St Lois MO) at a concentration of 75 µg/kg over a 48-hour period, as previously described [16]. Littermate controls were injected in parallel with the experimental animals. Bioluminescence imaging was performed 30 hours post injection.

### MRI

Anatomical MRI was performed using a 7T, 16 cm horizontal bore Agilent (Palo Alto, CA) *DirectDrive* system with a quadrature mouse head coil (m2m Imaging Corp., Cleveland, OH). Images were acquired twice weekly starting the day before treatment initiation. A T2-weighted spin-echo image was acquired for each mouse's right (tumor-bearing) leg using the following parameters: repetition time/echo time (TR/TE) = 4000/37 ms, field of view (FOV) = 20 mm, matrix size = 128×64, slice thickness = 0.5 mm and slice number = 25. Volumes of interest (VOI) were drawn manually on the T2-weighted images while regions of edema were omitted.

### Isolation of CD133 positive or negative GBM cells

D54-MG cells expressing the Caspase 3/7 GloSensor were stained using a CD133-specific primary antibody (Cell Signaling, Beverly, MA, USA) and a Cy5-labeled secondary antibody (Jackson Immunoresearch, St. Louis, MO, USA) and subsequently sorted using the BD FACS Aria system (BD, Franklin Lakes, NJ, USA). CD133 positive and negative cells were seeded 96-well white clear bottom plate (Corning, Inc., Corning, NY) 24 h prior to treatment with TRAIL (200 ng/ml), MK886 (50 µM), MNS (50 µM), and GW7647 (12.5 µM) and bioluminescence assays were performed as described above.

## Results

### Utility of Caspase 3/7 GloSensor for the assessment of cell death in cells

**Figure 1:**
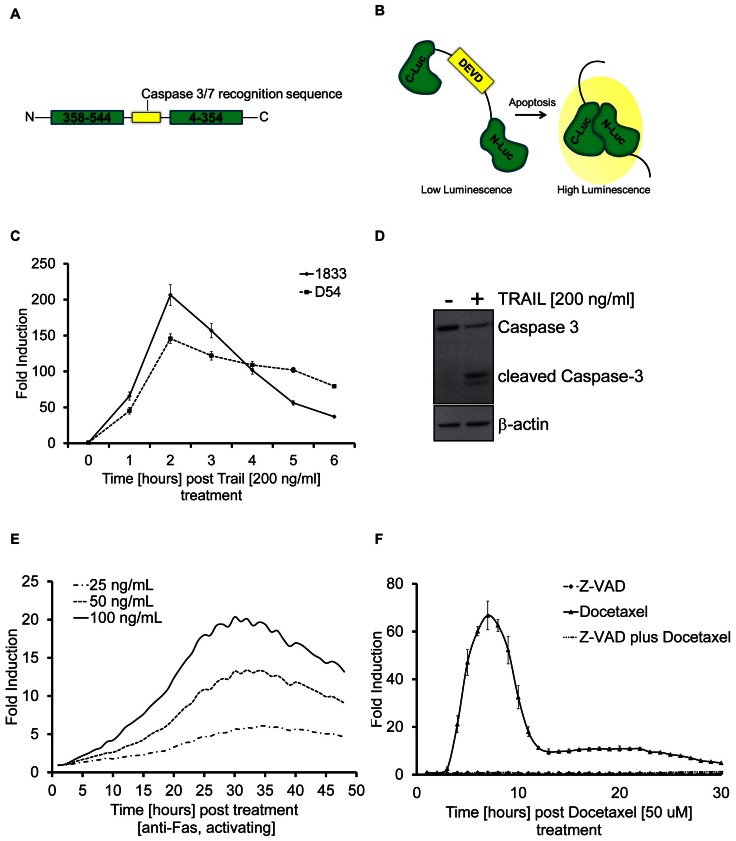
Utility of Caspase 3/7 GloSensor for assessment of cell death in cells. A) Schematic of the Caspase 3/7 GloSensor reporter containing an N-terminus coding for the C-Luc domain (358–544) of luciferase and a C-terminus coding for the N-Luc domain (4–354) of luciferase and a adjoining sequence, DEVD, the Caspase 3/7 recognition sequence. B) The functional basis of the reporter, wherein Caspase 3/7 mediated cleavage at the DEVD sequence results in release of the luciferase peptides and reconstitution of the enzymatic activity and an increase in luminescence signal. C) Bioluminescence analysis of cells treated with 200 ng/ml TRAIL. Data is plotted as fold induction standardized to values obtained from vehicle treated cells. D) Western blot for Caspase 3 cleavage using D54 cells treated with TRAIL for 6 hrs. β-Actin was used to standardize loading. E) Bioluminescence analysis of D54 cells treated with varying concentrations (25–100 ng/ml) of an agonist anti-Fas antibody. Data is plotted as fold induction over values obtained from vehicle treated cells at every hour. F) Bioluminescence analysis of cells treated with a pan-Caspase inhibitor Z-VAD (20 µM), 50 µM Docetaxel or with Z-VAD and Docetaxel combined. Data is plotted as fold induction. Experiments were performed at least in triplicates and mean values were plotted ± SEM.

We have developed a second generation reporter which detects the activation of Caspase 3 and 7 during apoptosis, Caspase 3/7 GloSensor. The reporter consists of an amino-terminal domain coding for the carboxyl-terminus of luciferase (C-Luc) encompassing amino acid 358–544 of *Photuris pensylvanica*. The carboxyl-terminus of the reporter codes for the amino-terminus of luciferase (N-Luc, residues 4–354) with an intervening caspase 3/7 recognition sequence, DEVD, (Figure 1A). Upon caspase 3/7 activation in response to apoptosis, as depicted in the schematic in Figure 1B, cleavage at the DEVD site enables reconstitution of luciferase, which results in an increase in bioluminescence activity.

To evaluate if the Caspase 3/7 GloSensor provides a surrogate for cell death in tissue culture and xenograft tumor models, cancer cell lines were generated which stably expressed the Caspase 3/7 GloSensor. In Figure 1C, the breast cancer line, 1833 and the glioma cell line D54 were treated with TRAIL a known inducer of Caspase 3 mediated apoptosis. Treatment of both cell lines resulted in a time and dose dependent increase of bioluminescence activity which correlated with the appearance of cleaved Caspase 3 as determined by western blotting analysis and depicted for D54 cells in Figure 1D. A dose dependent increase in bioluminescence activity was also observed using D54 cells in response to activation of the Fas death receptor using an agonist anti-Fas antibody (Figure 1E).

In contrast to TRAIL and Fas, which induce the extrinsic, receptor-mediated pathway of the apoptotic machinery, chemotherapy has been shown to induce Caspase 3 mediated cell death through the intrinsic pathway. To explore the utility of the biosensor in quantifiably measuring apoptosis through this pathway, we treated 1833 cells with 50 µM Docetaxel, a commonly used chemotherapeutic agent. In Figure 1F an increase of bioluminescence activity were observed upon Docetaxel treatment of 1833 cells, which was blocked in response to administration of a pan-Caspase inhibitor (Z-VAD), demonstrating the specificity of the reporter. Treatment of with Z-VAD alone did not result in any measurable bioluminescent signal.

### Caspase 3/7 GloSensor for measuring apoptosis in living animals

**Figure 2:**
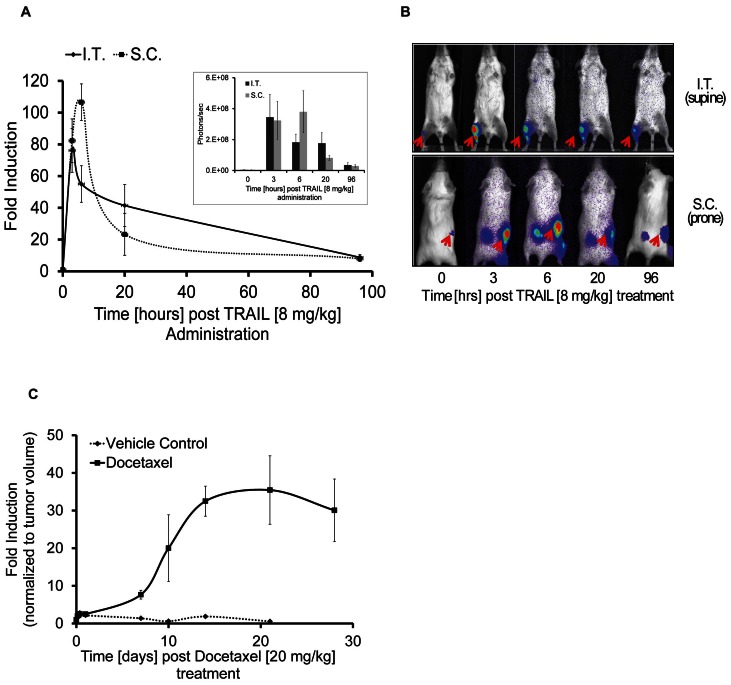
Detection of Apoptosis in mouse models using the Caspase 3/7 GloSensor. A) Fold induction of bioluminescence signal intensity over pretreatment values is plotted as mean ± SEM of both groups. At least four animals were used in each treatment group. Inset: Absolute bioluminescence signal (photons/sec) in mice containing subcutaneous (S.C.) or intra tibial (I.T.) tumors at 3, 6, 20, 96 hrs post TRAIL (8 mg/kg) administration. B) Representative bioluminescence images of animals are shown before treatment or at various time points post TRAIL administration. C) Tumor specific bioluminescence activity before treatment and at various time points post-treatment using 20 mg/kg Docetaxel, intravenously on day 7, 14 and 21. Fold induction of bioluminescence signal intensity over pretreatment values was plotted as mean ± SEM. for each group. At least four animals were used in each treatment group.

To assess whether this biosensor was useful for *in vivo* studies we implanted the 1833 cell line either subcutaneously into the flank or intra-tibially into SCID mice. Tumor growth was monitored by caliper (for S.C.) or MRI (for I.T.) measurements and tumors were allowed to reach 50–100 mm^3^ for S.C. and 10 mm^3^ for I.T. xenografts respectively. Luciferase activity was evalauted at 3, 6, 20 and 96 hours post TRAIL treatment. In control mice, luminescence values averaged approximately 4×10^6^ photons/second (SEM 1×10^6^) for animals with tumors in the tibia and 3×10^6^ photons/second (SEM 7×10^5^) for animals with subcutaneous tumors. Upon treatment with 8 mg/kg TRAIL, luminescence levels peaked at 3 hrs (3.5×10^8^ p/s (SEM 1×10^8^) and at 6 hrs (4×10^8^ p/s (SEM 1×10^8^) for animals with I.T. or S.C. tumors respectively (Figure 2A inset). As shown in Figure 2A a maximal fold induction of bioluminescence activity (80 or 100 fold) was reached after a single i.v. dose of 8 mg/kg TRAIL. Representative bioluminescence images of mice with either an intra-tibial tumor or a flank tumor upon TRAIL injection are shown in Figure 2B.

The reporter's utility for assessing cell death induced by chemotherapy was further demonstrate in an *in vivo* model of SCID mice bearing 1833 xenografts to the bone (I.T.) by repeated treatment of animals with 20 mg/kg Docetaxel at day 0, 7 and 14 (i.v.) (Figure 2C). Interestingly, the onset of apoptosis detection was delayed compared to TRAIL treatment and was seen in the second week of treatment, consistent with differences in the dynamics of induction of apoptosis by receptor mediated mechanisms compared to chemotherapy induced apoptosis.

**Figure 3:**
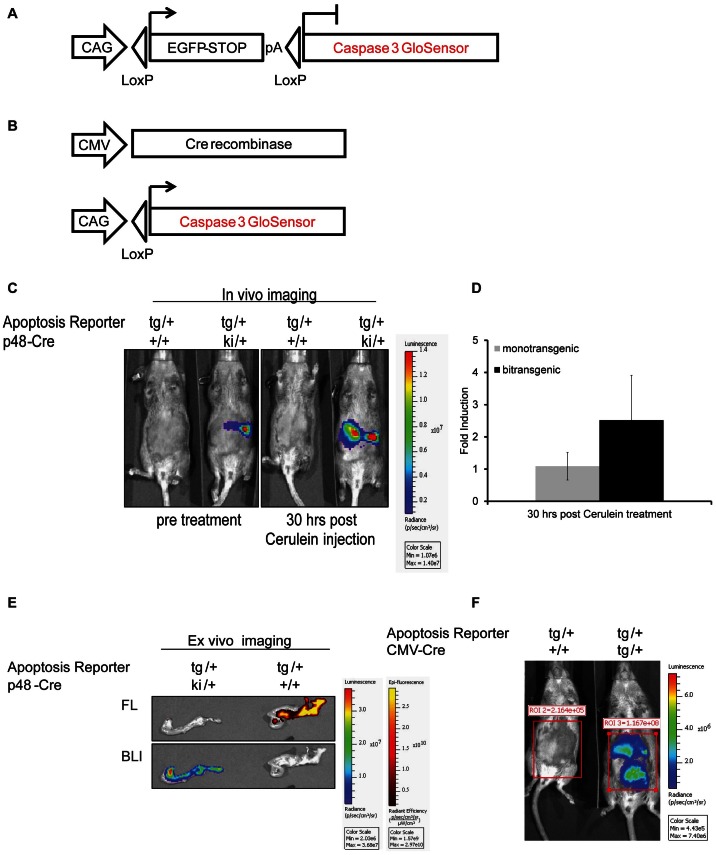
Transgenic reporter mice expressing Caspase 3/7 GloSensor. A) Schematic of the pCLEX Caspase 3/7 GloSensor transgene construct. B) Excision of the floxed EGFP-stop cassette when crossed with a Cre expressing mouse strain should result in tissue specific transcription of the reporter. C) Representative bioluminescence images of bi-transgenic (for the reporter and p48-Cre) or mono-transgenic (transgenic for the reporter in the absence of Cre) animals pre- and 30 hrs post-cerulein injection (75 ug/kg, total of 12 injections in 48 hrs). D) Quantification of BLI signal induction upon cerulein treatment. E) Representative bioluminescent and fluorescent (EGFP) ex-vivo images of pancreata from mono- or bi-transgenic animals. F) Representative bioluminescence images of bi-transgenic (right) or mono-transgic (left) animals.

Genetically engineered mouse models recapitulating a variety of human cancers are commonly used in the evaluation of therapeutics as co-clinical trials [17], [18]. To facilitate drug toxicity or efficacy studies we have engineered a mouse model wherein the GloSensor Caspase 3/7 reporter can be conditionally activated in a tissue specific manner to visualize apoptotic cell death in real-time and non-invasively. The transgenic reporter (Figure 3A) mice were crossed either with CMV-Cre (tissue non-specific) or p48-Cre (pancreas specific) expressing mice. Pancreas specific reporter expression was observed in littermates wherein Cre was expressed under the p48 promoter (tg/+, p48Cre ki/+), but not in mono transgenic animals (tg/+) (Figure 3C). Repeated administration of cerulein, an inducer of apoptotic cell death due to the induction of pancreatitis [19]–[21], was used to evaluate reporter activation. Caspase 3 activation was detected by western blot analysis in tissues derived from cerulein treated animals but not control animals (Figure S1 C). Luciferase activity was measured at 30 hours post-cerulein injection. In mono transgenic animals (absence of Cre expression), background luminescence values averaged 2×10^5^ photons/sec pre- and post-cerulein injection, whereas bi-transgenic animals showed bioluminescence values of 5×10^7^ photons/sec pre- and 1.5×10^8^ p/s post-treatment. As depicted in Figure 3C and 3D a 3–4 fold induction of bioluminescence activity was observed upon cerulein injection at 30 hrs post-cerulein injection. *Ex-vivo* fluorescence and bioluminescence imaging of the pancreas monotrasgenic for the Caspase3/7 reporter in the absence of Cre demonstrated the presence of EGFP expressing pancreatic cells (indicating that the lox-stop-lox cassette was present), yet were devoid of luciferase expression (Figure 3E). In contrast, pancreatic tissue from animals that were also transgenic for p48-Cre demonstrated bioluminescent activity as well as fluorescence, indicating Cre-mediated induction of the Caspase 3/7 GloSensor reporter (Figure 3E). Expression of the Caspase 3/7 GloSensor was achieved in multiple tissues by crossing the transgenic reporter mouse to a CMV-Cre deleter strain. As expected, bioluminescence activity was only detected in bi-transgenic animals (1×10^8^ photons/sec) but not in mice lacking Cre-expression (background levels of 2×10^5^ p/s, Figure 3F).

### Application of Caspase 3/7 GloSensor for high throughput screens

**Figure 4:**
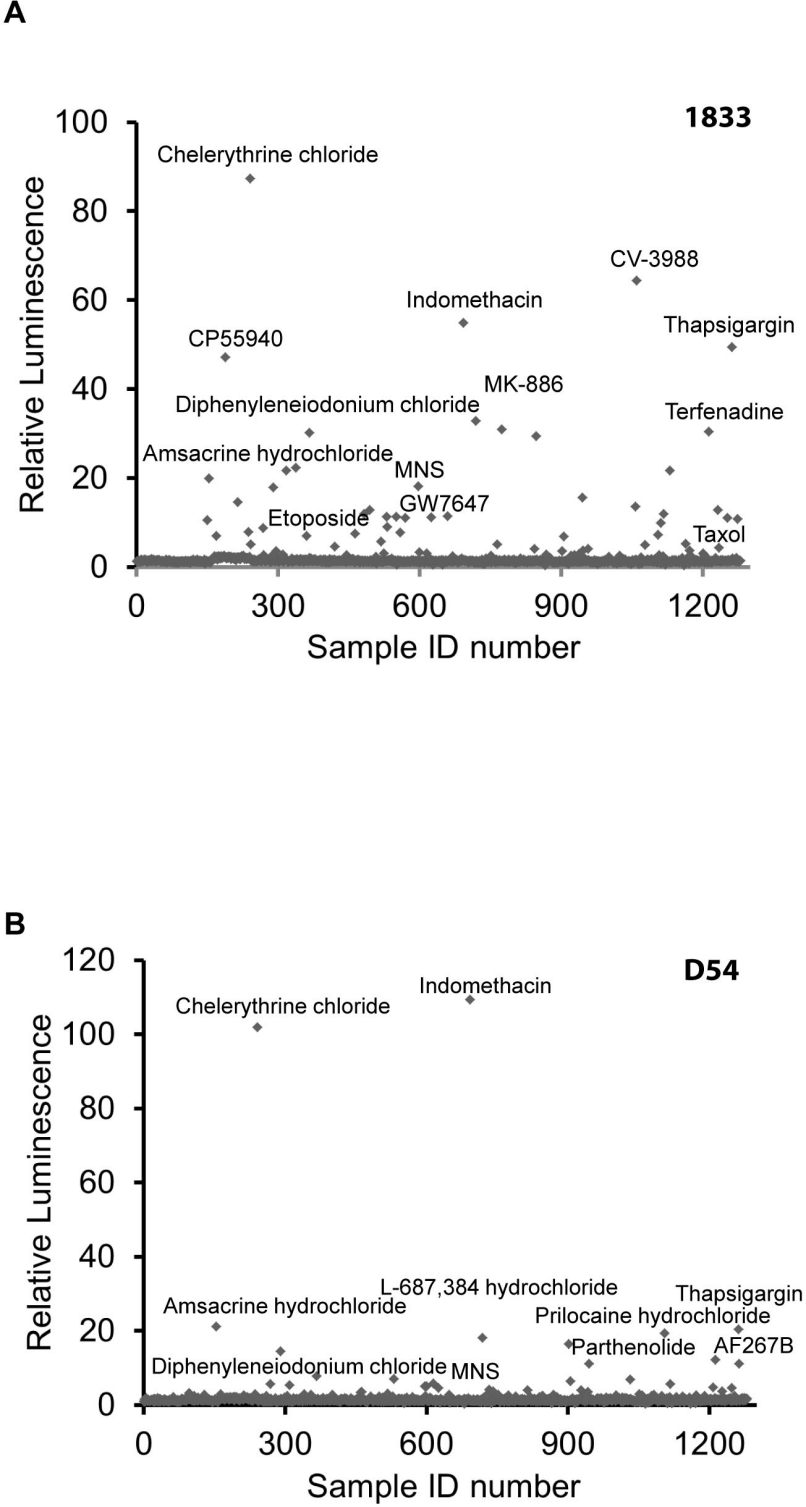
High throughput screening for inducers Caspase 3/7. Glosensor expressing 1833 or D54 cells (A and B respectively) were used to screen a library of 1,280 pharmacologically active compounds (LOPAC). Fold induction of bioluminescence signal intensity over values obtained from vehicle treated cells was plotted at maximal induction (mean ± SEM).

The high signal to noise and excellent dynamic range of the Caspase 3/7 GloSensor provided for an opportunity to screen for drugs in a high throughput format. The 1833 and D54 cancer cell lines, described above, were used for assay evaluation. Z-factors of >0.6 were achieved in 1833 and D54, respectively, upon treatment with TRAIL at various time points demonstrating the robustness needed for HTS. A library consisting of 1,280 pharmacologically active compounds (LOPAC) was used for screening the 1833 and D54 lines. The screen included intra plate negative and positive controls. The compounds were administered at 10 µM and bioluminescence activity was measured hourly over 24 hrs. Maximum fold induction of bioluminescence activity for the 1833 and D54 cell lines are shown in Figure 4A and 4B, respectively. Fold induction of above 3-fold was considered significant.

### Validation of HTS screen

**Figure 5:**
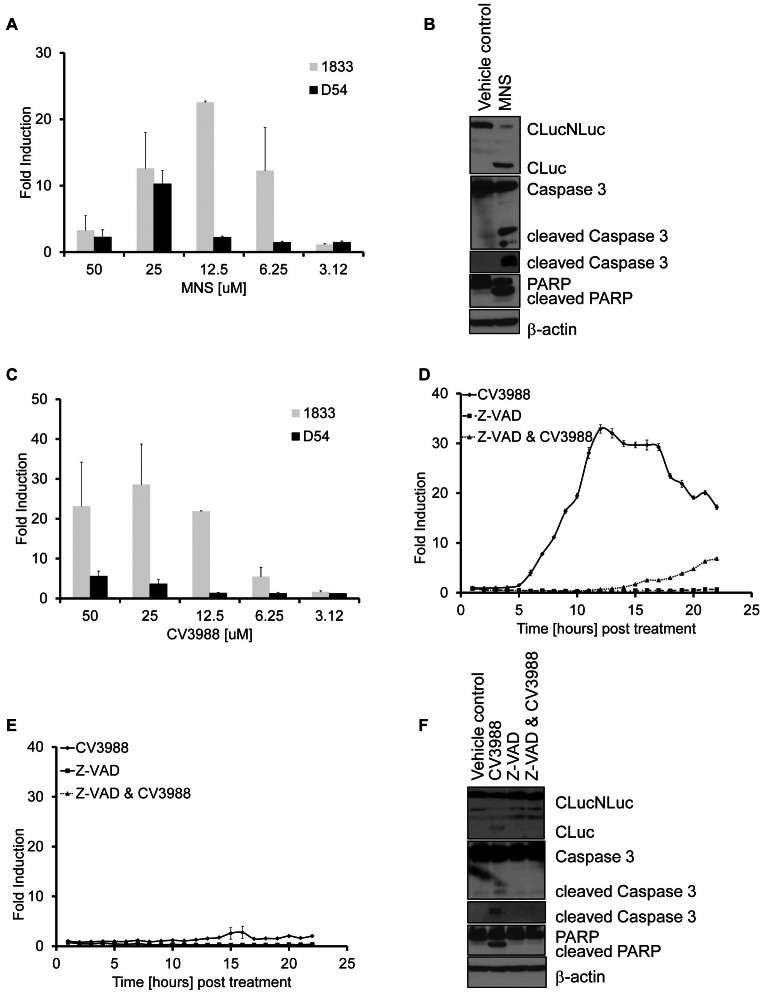
Validation of HTS hits. A) Bioluminescence activity of cells treated with MNS was measured at 12 hours post treatment and plotted as fold induction. Experiments were performed at least in triplicates (mean ± SEM). B) Representative western blots for Luciferase, cleaved Caspase 3 and PARP or β-Actin as loading control of D54 cells treated with (25 µM) MNS for 12 hrs. C) Bioluminescence activity of cells treated with increasing concentrations of CV3988 at 12 hours post treatment. Data are plotted as fold induction over values obtained from vehicle treated cells. Experiments were performed in triplicates (mean ± SEM). D and E). Bioluminescence activity was measured at various time points using 1833 (D) or D54 (E) cells treated with CV3988 (12.5 µM), Z-VAD (20 µM) or a combination of Z-VAD plus CV3988 for 24 hrs. Data are plotted as fold induction and experiments were performed in triplicates and plotted as mean ± SEM. F) Representative western blots of Luciferase, Caspase 3, PARP or β-actin were performed on lysates obtained from D54 cells. Cells were either treated with CV3988 (12.5 µM), pre-treated with Z-VAD (20 µM) or treated with Z-VAD and CV3988 for 12 hrs.

Selected hits from the HTS screen were further evaluated for the induction of cell death. MNS, a Src kinase inhibitor, induced Caspase 3 reporter activation in both cell lines although 1833 cells were more sensitive compared to D54 (Figure 4A). MNS induced Caspase 3 and PARP cleavage, a Caspase 3 substrate as assessed by western blotting (Figure 5B) of lysates obtained from D54 cells treated with MNS for 3 hrs. Interestingly, we also identified compounds, such as CV3988 which were highly selective for one cancer cell line but failed to induce measurable amounts of Caspase activation in the other evaluated cell line. As shown in Figure 5C, induction of Caspase 3 activity was observed in 1833 at varying concentrations of CV3988, yet activity in D54 remained negligible even at higher doses (Figure 5C and 5F). To confirm that reporter activation in 1833 cells was due to Caspase 3 activation, cells were pre-treated with the pan-Caspase inhibitor Z-VAD. As shown in Figure 5E, Z-VAD completely attenuated the induction of bioluminescence activity upon CV3988 exposure. Activation of Caspase 3 and its downstream target (PARP) as well as Z-VAD mediated inhibition was confirmed by western blot analaysis (Figure 5D).

### Identification of intrinsic differences in the apoptotic machinery between stem and non stem cells

**Figure 6:**
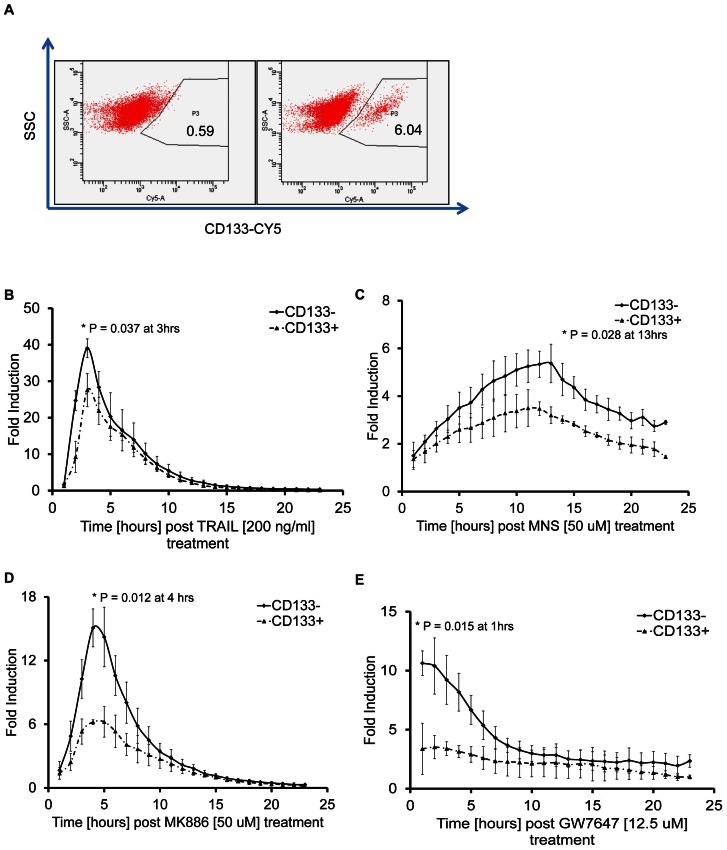
Assessing drug sensitivity of rare and transient cell populations. A) FACS analysis of dissociated D54 cells sorted into CD133^+^ and CD133^−^ populations, P3 represents the CD133 expressing cell population. B) to E) Bioluminescence assay of CD133^+^ and CD133^−^ sorted D54 cells incubated with 200 ng/ml TRAIL (B), 50 µM MNS (C), 50 µM MK886 (D) or 12.5 µM GW7647 (E). Bioluminescence was plotted as fold induction over values obtained from vehicle treated cells. Experiments were performed in triplicates and plotted as mean ± SEM. Paired t-test was performed for all experiments and * denotes p<0.05 value at indicated time points.

We evaluated the suitability of the reporter for imaging apoptosis in rare and transient cell populations, such as cancer stem cells, which have self renewal properties and are often found to contribute to therapeutic resistance. The glioma line D54 has previously been shown to contain a small (<10%) population of cells expressing the stem cell marker CD133. Thus D54 cells, expressing the Caspase 3/7 GloSensor were FACS sorted into CD133 positive and CD133 negative subpopulations (Figure 6A). Although both populations responded to TRAIL with an increase in bioluminescence activity, CD133-positive cells showed a relative resistance to TRAIL as seen by a significantly lower induction of bioluminescence activity (Figure 6B). To test whether this response was TRAIL specific or indicative of intrinsic differences in the apoptotic machinery between CD133-positive and negative cells, compounds identified as Caspase 3 inducers in the HTS were evaluated. Activation of the reporter in response to treatment with MNS, MK886 and GW7647 was found to be significantly lower in CD133-positive cells compared to the CD133-negative population suggesting that the stem-like phenotype included inherent resistance to apoptosis (Figure 6C–E).

## Discussion

Evaluation of new therapeutics and combination therapies for cancer requires the use of biological assays that allow for rapid and specific evaluation of efficacy in cell based assays and in animal models [1]. While some methods are better suited for HTS than others, very few can be translated into *in vivo* systems. In addition, clinical data has shown that it is becoming increasingly important to distinguish between different mechanisms of cell death [1] and therefore development of an apoptosis surrogate which can be used to identify novel therapeutics in intact cells with subsequent evaluation using *in vivo* models would be useful and important for streamlining the drug discovery and drug development process.

Here we described a bioluminescent apoptosis surrogate for use in HTS of therapeutics which is also capable of being used for *in vivo* imaging of apoptotic cell death. Recently, protease biosensors have been developed using a circularly permuted form of firefly luciferase [5], [10]. In these studies, the authors created multiple protease biosensors by fusing the N- and C-termini of *Photinus pyralis* luciferase with a short peptide containing a protease cleavage site, where new termini were created elsewhere in the structure via circular permutation. These constructs showed a large increase in luminescence following protease cleavage of the peptide linker. Although this approach gave robust results in an *in vitro* enzyme assay, reduced responsiveness was seen when a subset was tested in living cells grown in culture. We hypothesized that once cleaved, the resulting luciferase fragments might easily dissociate in the absence of tightly interacting peptides [6] or covalent association [22]. In the context of a living cell, these fragments may also be susceptible to proteolysis, limited enzymatic half-life or non-productive interactions with cellular components, such as protein chaperones. To address this, we extended the current approach to a thermal stable luciferase [11] permuted at residue 233 or 358. When used to create caspase-3/7 sensors, both constructs showed robust induction following treatment with TRAIL, where we hypothesized that the cleavage fragments were better able to associate given the increased stability inherent within the overall structure. In an attempt to further improve response dynamics, we performed random mutagenesis using the 358 construct and screened for mutants showing increased fold response both *in vitro* and in cell culture. The best performing construct, 358V3 was found to contain a single Thr to Ile point mutation, which provided a 3–4 fold higher increase in luminescence compared to the parent 358 construct.

To evaluate the improved reporter for imaging of apoptosis in cell culture, the 1833 breast and D54 glioma cell lines were engineered to stably express the reporter. The high signal to noise ratio observed following treatment with TRAIL, Docetaxel and other compounds was attributed to the improved structural stability of the engineered protein (Figure 1–3). The ability to non-invasively image apoptosis in a dynamic manner allows for rapid evaluation of therapeutic efficacy. As little as 3 hrs post-therapy an apoptotic response was detected. Importantly, pre-treatment of cells with Z-VAD, a pan-Caspase inhibitor abrogated the induction of bioluminescence thus confirming the specificity of this reporter to Caspase activation (Figure 1F and 5E&F). In the *in vivo* setting, TRAIL or Docetaxel treatment of xenograft tumors showed high levels of reporter activation which correlated with Caspase 3 activation, thereby confirming the robustness of the reporter for imaging cell death noninvasively and dynamically over time in living animals. Importantly, the reporter's utility was extended to detection of apoptosis in response to chemotherapeutic intervention. This we believe would provide an opportunity to rapidly and quantitatively translate new therapeutic discoveries from cells to mouse models and also enable optimization of dosing and schedule optimization of therapeutics.

Mouse models of cancer provide for a more clinically relevant model system compared to xenografts and therefore, a genetically engineered mouse has been developed wherein Caspase 3/7 GloSensor expression can be achieved upon Cre-dependent ablation of a lox-STOP-lox cassette. Application of this engineered mouse would be widely applicable across a variety of human diseases including cancer, neurodegeneration (i.e. Alzheimer's & Parkinson's) and inflammation. The ability to activate the reporter in a tissue specific manner (e.g. tumor cells as demonstrated here, or neurons and inflammatory cells) provides this versatility. Additionally, activating reporter expression in multiple tissues by crossing the mouse to Cre-deleter strains, such as CMV-Cre or Rosa26-Cre, represents an opportunity for assessment of normal tissue toxicity in response to therapeutic intervention.

In addition we also demonstrate the applicability of the Caspase 3/7 GloSensor for HTS of anticancer agents using apoptosis as an efficacy readout. The optimized reporter structure lends itself to quantification of compound efficacy dynamically over time rather then many current assays which provide for a end-point readout. This feature can, in principle, provide for more optimal detection of ‘hits’ by allowing for the maximal signal to be detected as different compounds are likely to have differing temporal effects on cell death. For example, one compound might induce cell death within minutes and at very low concentrations whereas other compounds might induce maximal activation of the cell death machinery many hours later. Evaluation of this reporter was accomplished in breast and glioma cancer lines against a library of 1,280 pharmaceutically distinct compounds revealing that Caspase induction could be monitored and quantified over a period of at least 24 hrs without the need of repeated adminstration of the substrate, luciferin. Moreover, in this relatively small scale validation study of our reporter using 1,280 compounds, we were able to demonstrate that specific compounds were effective Caspase 3 activators in both cell lines whereas other compounds were found to be active in only one of the lines. For example, the drug Chelerythrine chloride, which is a known apoptosis inducer by displacing BAX binding and subsequent inhibition of BclXL function, induced a very strong activation of the apoptosis reporter in our HTS in both cell lines used [23]. The non-steroidal anti-inflammatory indomethacin also induced apoptosis in both lines. Interestingly, CV3988, a competitive platelet activating factor (PAF) receptor antagonist, selectively induced apoptosis in 1833 cells, indicating a context dependent activation of the apoptotic machinery (Figure 5D &5E). Validation of the HTS hits demonstrates the specificity of the reporter in a HTS platform. Although, the screen was conducted at 10 µM, MNS showed significant efficacy at doses as low as 6 µM. Thus, differential sensitivity and specificity of MNS, as a selective Src inhibitor, was recapitulated in a non-HTS assay in 1833 cells. Interestingly, efficacy of Src-inhibition in preventing bone metastasis of breast cancer using the 1833 cells was recently demonstrated, validating our findings using this apoptosis reporter technology [24].

Intrinsic or acquired drug resistance represents a re-occurring obstacle in the effective treatment of cancer patients. Here we demonstrated that intrinsic differences in response to therapy a subpopulation of cells can be evaluated using the GloSensor technology. D54 cells expressing the stem cell marker CD133, had an intrinsic resistance to a variety of compounds confirming prior findings [25], [26]. The reporter's exceptional sensitivity lends it to evaluation of therapeutic efficacy in rare cell populations (less than 500, see Figure S1), which is particular important when these populations are transient in nature, as has been demonstrated for CD133 positive cancer stem cells.

Overall, the results presented in this study reveal that our novel Caspase 3/7 GloSensor is suitable for use in high throughput cell-based screening assays thus allowing for rapid identification of efficacious compounds. Furthermore, the ability to rapidly adapt the assay for *in vivo* application should facilitate studies of toxicity, drug-target interactions, optimization of dose and schedule and utility in co-clinical trials. Lastly, the development of genetically engineered mouse models wherein expression of the Caspase 3/7 GloSensor can be achieved in a tissue specific manner provides a unique opportunity to evaluate the role apoptoses (as well as other proteolytic events in the future) using mouse models of human disease.

## Supporting Information

Figure S1 **Detection of Caspase 3 activation in rare cell population.** A) & B) Bioluminescence assay in D54 (A) or 1833 (B) cells using as little as 100 cells per well. Cells were treated with 200 ng/ml TRAIL. Data are plotted as fold induction over values obtained from vehicle treated cells. C) Cleaved Caspase 3 Western blot of pancreatic tissue samples obtained from animals left untreated or treated with cerulein. β-Actin western blotting was used to confirm equal loading.
(JPG)
