# Supplementary figures and images for: Imaging Proteolytic Activity in Live Cells and Animal Models
Source: PLoS One. 2013 Jun 11;8(6):e66248. doi: 10.1371/journal.pone.0066248 (PMC3679058; doi:10.1371/journal.pone.0066248)

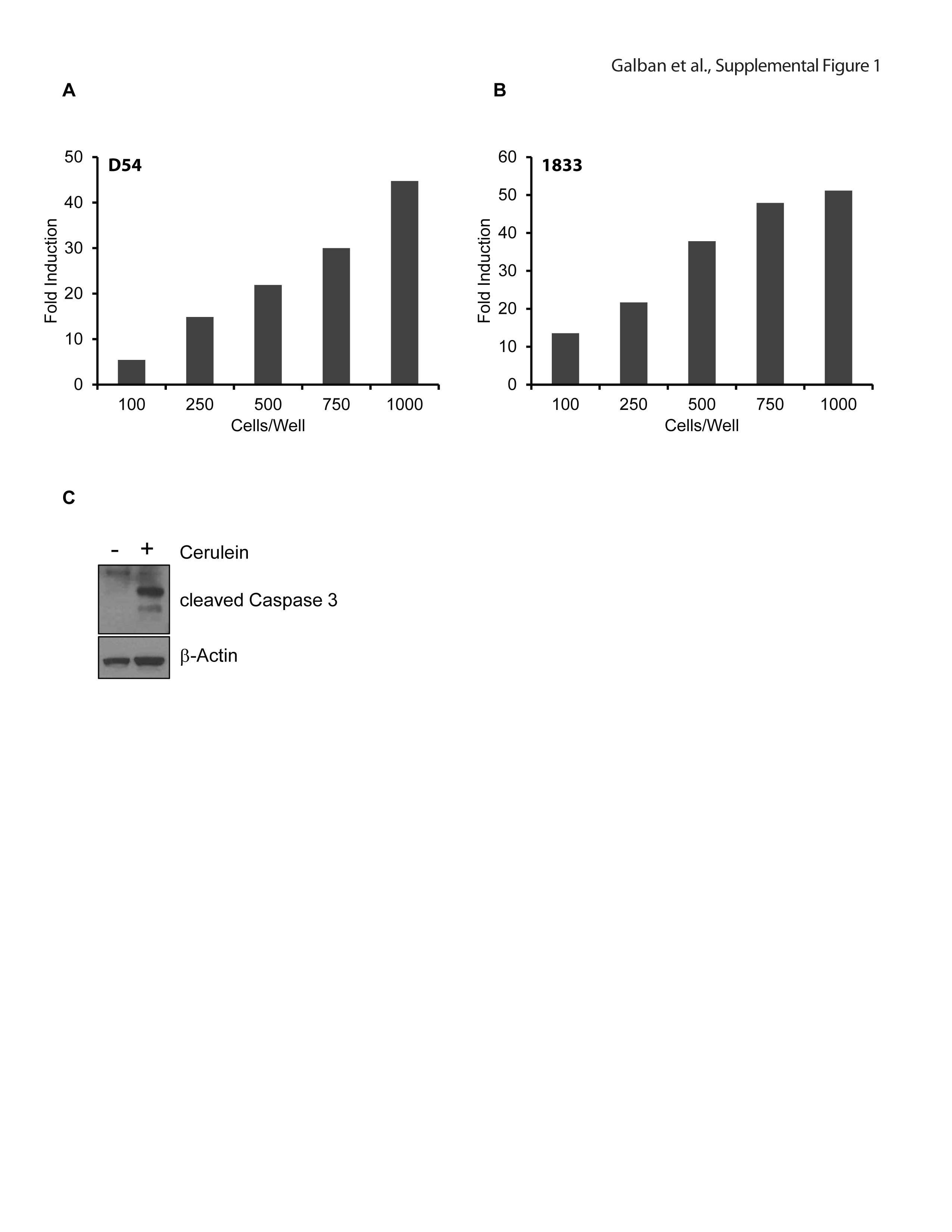

Supplement: Figure S1 — Detection of Caspase 3 activation in rare cell population. A) & B) Bioluminescence assay in D54 (A) or 1833 (B) cells using as little as 100 cells per well. Cells were treated with 200 ng/ml TRAIL. Data are plotted as fold induction over values obtained from vehicle treated cells. C) Cleaved Caspase 3 Western blot of pancreatic tissue samples obtained from animals left untreated or treated with cerulein. β-Actin western blotting was used to confirm equal loading. (JPG) [file pone.0066248.s001.jpg]
